# Optimal combination of MYCN differential gene and cellular senescence gene predicts adverse outcomes in patients with neuroblastoma

**DOI:** 10.3389/fimmu.2023.1309138

**Published:** 2023-11-16

**Authors:** Jiaxiong Tan, Chaoyu Wang, Yan Jin, Yuren Xia, Baocheng Gong, Qiang Zhao

**Affiliations:** ^1^Tianjin Medical University Cancer Institute and Hospital, National Clinical Research Center for Cancer, Tianjin, China; ^2^Tianjin’s Clinical Research Center for Cancer, Tianjin, China; ^3^Key Laboratory of Cancer Prevention and Therapy, Tianjin, China; ^4^Department of Pediatric Oncology, Tianjin Medical University Cancer Institute and Hospital, National Clinical Research Center for Cancer, Tianjin, China

**Keywords:** neuroblastoma, cellular senescence, tumor microenvironment, COLD TUMOR, prognosis

## Abstract

**Introduction:**

Neuroblastoma (NB) is a common extracranial tumor in children and is highly heterogeneous. The factors influencing the prognosis of NB are not simple.

**Methods:**

To investigate the effect of cell senescence on the prognosis of NB and tumor immune microenvironment, 498 samples of NB patients and 307 cellular senescence-related genes were used to construct a prediction signature.

**Results:**

A signature based on six optimal candidate genes (TP53, IL-7, PDGFRA, S100B, DLL3, and TP63) was successfully constructed and proved to have good prognostic ability. Through verification, the signature had more advantages than the gene expression level alone in evaluating prognosis was found. Further T cell phenotype analysis displayed that exhausted phenotype PD-1 and senescence-related phenotype CD244 were highly expressed in CD8+ T cell in MYCN-amplified group with higher risk-score.

**Conclusion:**

A signature constructed the six MYCN-amplified differential genes and aging-related genes can be used to predict the prognosis of NB better than using each high-risk gene individually and to evaluate immunosuppressed and aging tumor microenvironment.

## Introduction

Neuroblastoma (NB) is the most common pediatric solid tumor, but it poses a challenge in terms of treatment. Approximately half of the patients are diagnosed with high-risk NB and undergo intensive multimodal therapy, yet the 5-year overall survival (OS) rate remains below 20% (1). The occurrence and development of most tumors are closely related to the MYC gene family, and one of the well-known MYC genes is involved in proliferation, apoptosis, and differentiation are also associated with PD-L1 expression (2). MYCN, another member of the MYC gene family, is mainly involved in nervous system development and tumor formation (3). Prognosis in NB is known to be influenced by factors such as age, tumor cell differentiation, and MYCN amplification, but the MYCN gene itself is not easily targeted therapeutically (4). While tumor-specific monoclonal antibodies (mAbs) targeting GD2 have become a standard component of therapy for high-risk NB patients, the risk of relapse remains high. This highlights the potential for immunotherapeutic approaches to reduce the risk of recurrence (5).

Immune checkpoint inhibitors (ICIs) therapy, which enhances certain aspects of the immune system to recognize and eliminate tumor cells, has shown efficacy in several solid tumors but has limited curative effect in NB (6). In 2009, Camus et al. first classified tumors into “cold” and “hot” based on the distribution of immune cells, particularly T lymphocytes, and their differential responses to immunotherapy (7). NB is considered a good experimental model for studying immunotherapy resistance, as it is a cold tumor and presents an opportunity to investigate strategies to transform it into a hot tumor to improve the efficacy of immunotherapy (8). In our preliminary study, we observed that Anlotinib, an orally administered small-molecule multitarget tyrosine kinase inhibitor, induced a T cell inflamed tumor microenvironment (TME) by facilitating vessel normalization, thereby enhancing the efficacy of PD-1 checkpoint blockade in NB (9). Additionally, NB tumor cells have a low mutation load and lack major histocompatibility complex I (MHC-I) expression, which contributes to their low immunogenicity and prevents T cells from recognizing them (10). The bromodomain and extra-terminal domain (BET) protein family mediates T cell exhaustion and hinders proliferation and differentiation of NB cells (11, 12). However, the above description does not fully explain the immune escape mechanism of NB or provide a comprehensive understanding of the differential expression of multiple immunosuppressive receptors. Senescence was first described by Hayflick and Moorhead in 1961 based on observations of *in vitro* cultured human fibroblasts. It refers to the loss of proliferative potential in cells after a defined number of passages (13). Under normal circumstances, senescent cells undergo a permanent cell-cycle arrest but remain metabolically active in the G0 phase, with physiological implications for cellular metabolism. However, recent observations have shown that senescent cells can reprogram into a stem cell state and re-enter the cell cycle in tumor mice (14). When chemotherapy-induced senescence therapy is discontinued, tumor cells can exit the senescent state and even resume enhanced growth (15). Cellular senescence is a complex adaptive process that involves the expression of senescence-related secretory phenotype (SASP) and the release of cytokines and growth factors, contributing to tumor immune escape and progression (16). One crucial aspect of tumor immune escape and targeted cell cycle drug killing is that tumor cells enter the G0 phase by expressing a senescent phenotype to evade recognition and clearance by the immune system and chemotherapy drugs (17). Importantly, senescence-related genes are significantly associated with adverse clinical outcomes in various cancers, providing valuable insights for risk stratification and understanding the immunosuppressive tumor microenvironment (18, 19).

In this study, we investigated the effects of senescence gene combinations on outcomes and the immune microenvironment in NB by screening differential genes between MYCN-amplified and non-MYCN-amplified NB samples. Ultimately, we successfully constructed a prognosis prediction signature of NB based on six genes. Validation experiments revealed that the score calculated by this signature closely predicted prognosis compared to the relative expression of a single high-risk gene. Our findings were further validated using NB cell line and *in vitro* co-culture experiments. The results demonstrated that different tumor antigens influenced the distribution of T cell subsets, with the depletion phenotype PD-1 and cell senescence phenotype CD244 overexpressed in CD8+T cell subsets.

## Methods

### Data source

The gene expression profiles and clinical information from 498 primary NBs were obtained from the Gene Expression Omnibus (GEO) database, specifically the GSE49710 dataset, using RNA-Seq and microarrays. The GEO database can be accessed at https://www.ncbi.nlm.nih.gov/geo/. To investigate cellular senescence in NB, we utilized a list of 307 cellular senescence-related genes downloaded from the Cell-Age database. This database can be found at https://genomics.senescence.info/cells/. For external validation of our signature, we utilized the E-MTAB-8248 dataset from the ArrayExpress database. This dataset consists of 223 samples and can be accessed at https://www.ebi.ac.uk/biostudies/arrayexpress.

### Building and verification of the signature

**Figure 1:**
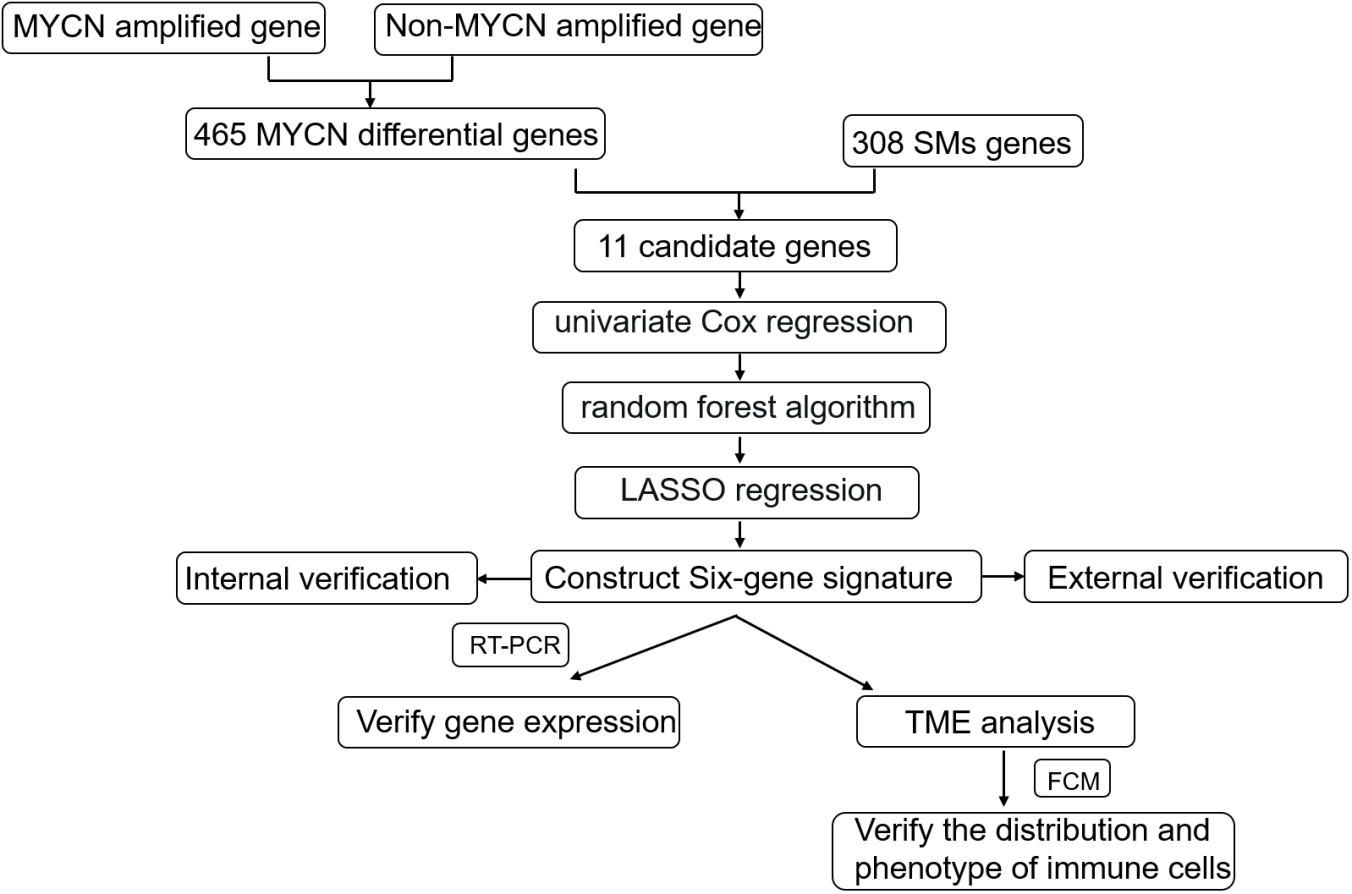
Schematic diagram of the study design. Gene expression and clinical information from 498 primary NBs were obtained from the GSE49710 dataset and internal verification through the same database;308 cellular senescence-related genes were obtained from the Cell-Age database; 223 sample data from the E-MTAB-8248 dataset were used for external validation. The internal verification included the relationship between 11 candidate genes and NB prognosis, the relationship between the constructed signature and NB prognosis, and the relationship between age, INSS stratification, clinical risk, and MYCN status; External validation focused on the relationship between signature and NB prognosis and the fit degree of prognosis prediction.

The 498 samples from the GSE49710 dataset were used for signature development, and samples from E-MTAB-8248 were used for model validation. Firstly, we identified 476 differentially expressed genes (DEGs) using the R package “limma”. Then, we performed univariate Cox analysis to identify the intersection of differential genes and cellular senescence-related genes. Next, we analyzed the relationship between the selected overlapping genes and the prognosis of NB patients using Kaplan-Meier (K-M) survival statistics. To further screen the DEGs, we employed random forest analysis and least absolute shrinkage and selection operator (LASSO) regression analysis. Using mean decrease accuracy and mean decrease gini coefficients, we identified the top six genes (TP53, IL-7, PDGFRA, S100B, DLL3, and TP63) with the highest coefficients. LASSO assigned regression coefficients to each gene and combined them into an algorithmic model. The predictive performance of the model was assessed using Kaplan-Meier analysis and the area under the curve (AUC) of the receiver operating characteristic (ROC) curve. The above processes were shown in **Figure 1**.

### Analysis of immune microenvironment and tumor cell stemness

To assess the level of immune infiltration, we employed four algorithms: “Estimation of Stromal and Immune cells in Malignant Tumors using Expression data (ESTIMATE)” (20), “Cell-type Identification By Estimating Relative Subsets Of RNA Transcripts (CIBERSORT) (21) ”, “Microenvironment Cell Populations-counter (MCP-Counter) (22)”, and “xCell” (23). Then the correlation between the risk score and tumor cell stemness was analyzed. All the data analyzed in this study were obtained from the GSE49710 dataset.

### Cell culture system *in vitro*

Peripheral blood mononuclear cells (PBMCs) were isolated using lymphocyte separation solution. First, the peripheral blood of volunteers was diluted 1:1 with PBS, and then the diluted blood was spread on 4ml lymphocyte separation solution and centrifuged for 15 minutes at 1500rpm. Then, the cells in the suspended particle layer were absorbed by a glue head dropper and transferred to a centrifuge tube equipped with PBS, mixed and cleaned, and centrifuged at 1000rpm for 10 minutes. The PBMCs were then plated in a petri dish at a concentration of 1x10^6 cells. The culture medium consisted of a mixture of 1640 and 10% fetal bovine serum at a ratio of 9:1. To activate the T cells, IL-2 (at a concentration of 1000u/mL) and CD3/CD28 antibodies were added to the cell culture as per the instructions provided. SK-N-BE (2) (MYCN-amplified) and SH-SY5Y (non-MYCN amplified) cell lines as different tumor antigens were selected. DMEM/F12 was used as a culture medium for cell lines, and 10% fetal bovine serum (FBS) and 1% Penicillin/Streptomycin were added, pre-heated to 37°C, and sterilized by filtration under sterile conditions. The cell suspensions are transferred to sterile cell culture vials, with enough medium added to each vial to make the cell density appropriate (usually 70-80% bottle surface coverage). The cell culture vial is placed in a cell incubator at 37°C to provide the appropriate temperature and CO2 concentration (5%) and the medium is changed every two to three days to maintain healthy cell growth. These cell lines were chosen to ensure that T cells could be fully exposed to the tumor antigens and evaluate the impact of the tumor microenvironment (TME) on T cell responses.

### Real-time quantitative PCR

The sequence of primers associated with each gene for qPCR is provided in **Supplemental Table 1** (submitted). SK-N-BE (2) cell lines and SH-SY5Y NB cell lines were amplified using conventional cell culture methods. Briefly, mRNA was extracted using a commercial kit (Total RNA Purification Kit, NORGEN) and quantified using the spectrophotometer (Thermo Fisher Scientific). The mRNA was reverse transcribed in cDNA using the High-Capacity RNA-to-cDNA™ Kit (Applied Biosystems™), and qPCR was carried out using SYBR green PCR master mix (Applied Biosystems™) in Bio-Rad CFX Manager.

### Flow cytometry

To detect the proportional and phenotypic changes in T cell subsets, cell surface staining analysis was conducted using multi-colored fluorescent flow cytometry. The following antibodies were used: CD3-FITC (clone HTT3a), CD8-Cy5.5 (clone SK1), CD244 (2B4)-PE (clone C1.7), CD4-APC-H7 (clone RPA-T4), PD-1-PE-CY7 (clone A17188B), PE-CY7-isotype control (clone MPO-11). These antibodies were ordered from BD Biosciences (San Jose, USA) and Biolegend. First, PBMC after exposure to tumor antigen were centrifuged for standby staining. Then, fluorescent monoclonal antibodies were added to the cells and incubated in the dark at room temperature for 15-20 minutes. The samples were washed twice with 1x PBS to remove excess fluorescent antibodies and broken cell fragments. Fully viable cells were acquired for analysis using a BD FACS-CantoIIflow cytometer (BD Biosciences, San Jose, USA), and subsequent analysis was performed using Flowjo software (Flowjo LLC, USA). Prior to obtaining the target cells, dead and sticky cells were subsequently excluded using FSC-A/FSC-H.

### Statistical methods

Statistical analysis and graphing were performed using the R software (version 4.2.1) and GraphPad Prism 8. To compare differences between two groups, we employed two-tailed unpaired Student’s t-test and Wilcoxon test. The Fisher’s exact test was used to compare differences in qualitative variables between the two groups. Statistical significance was defined as P < 0.05.

## Results

### Screened target associated with NB prognosis from MYCN amplified differential genes and senescence molecules (SMs) gene

**Figure 2:**
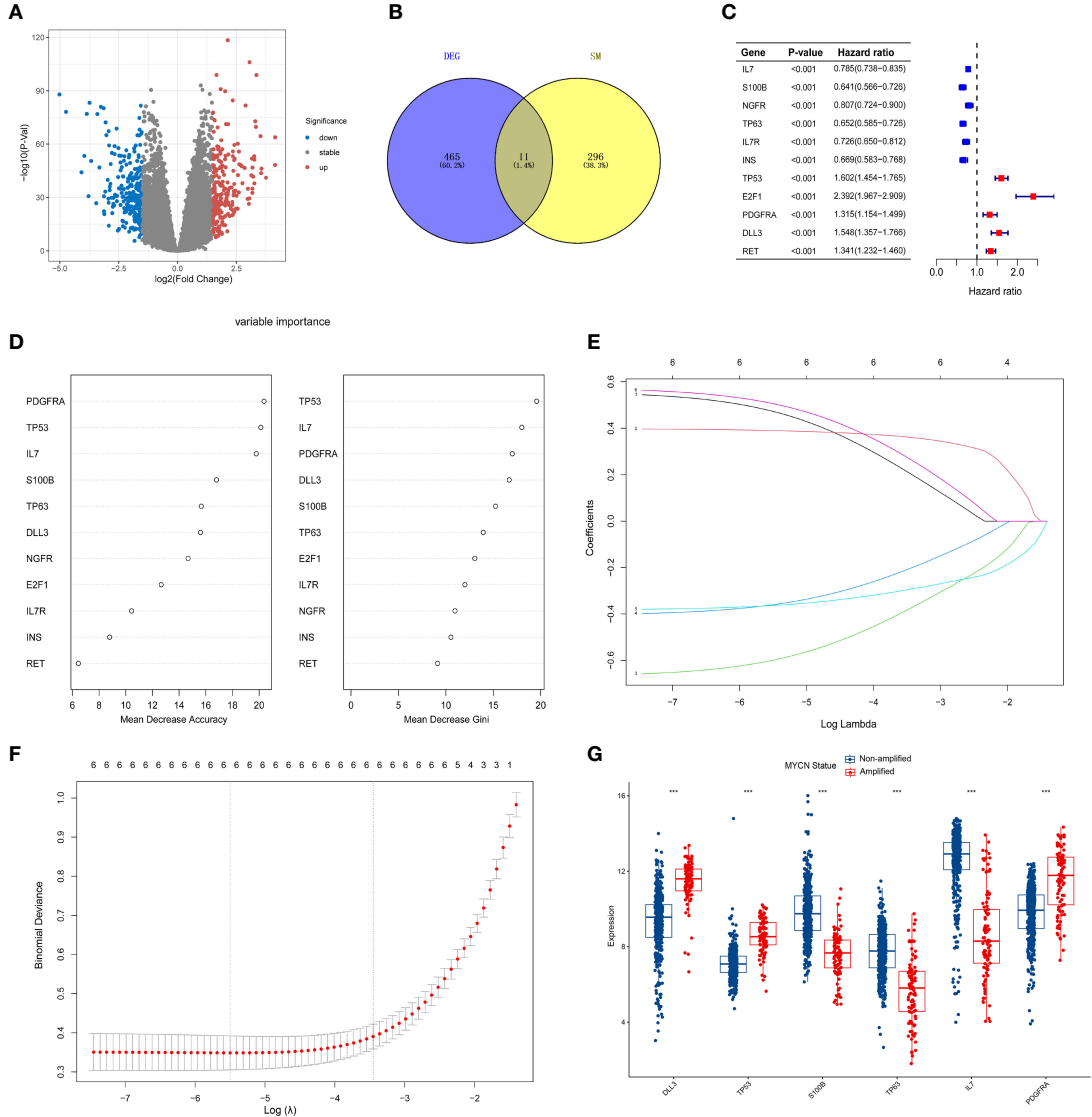
Screening of candidate genes and construction of signature **(A)** The volcano plot for differentially expressed genes (DEGs) (|log2FC| > 1.5 and adjusted *p* < 0.05), and the red, gray and blue circles indicate up- regulated, stable expressed and down-regulated of MYCN genes, respectively. **(B)** The blue regions represent 465 MYCN-amplified differential genes, while the yellow regions represent 308 SMs genes. **(C)** Forest diagram displaying the univariate Cox proportional hazard regression model for eleven genes and all candidate genes were associated with poor OS in GSE49710 datasets. **(D)** random forest algorithm results of 11 genes. **(E, F)** Results of LASSO regression analysis of the top six genes. **(G)** Differences between the MYCN amplified and non-amplified groups of six candidate genes and the risk-score signature constructed based on the candidate genes. ***p < 0.01.

MYCN amplification in NB patients has been identified as a poor prognostic factor (4). Therefore, it is crucial to first screen for MYCN-related differential genes. After downloading the GSE49710 dataset, we preprocessed the expression profile data and identified differentially expressed genes (DEGs). The volcano plot for the DEGs is presented separately in **Figure 2A**. 465 differential genes met the criteria for further analysis (|log2 fold change (FC)| > 1.5 and adjusted p-value < 0.05). To identify the intersection between MYCN-amplified differential genes and 307 SMs genes, we identified eleven genes, as shown in **Figure 2B**. Subsequently, we included these eleven identified genes in the univariate Cox regression model to analyze their relationship with prognosis. The results demonstrated that all genes were significantly associated with poor OS in NB patients in the GSE49710 dataset, as depicted in **Figure 2C** (p-value < 0.001).

### Construction of the six-gene signature

**Figure 3:**
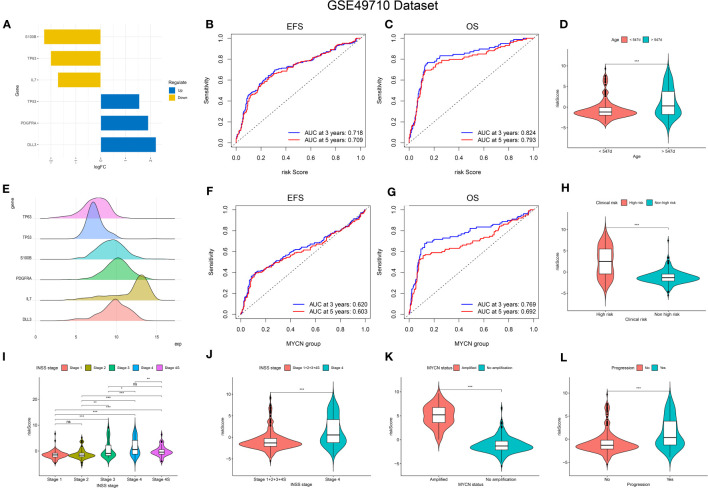
Relationship of signature with age, MYCN-status, clinical prognosis, and INSS grading. **(A, E)**, Expression trends and amounts of six genes included in the model; **(B, C, F, G)**: the ROC curve of the signature and MYCN status regarding EFS and OS; **(D, H, I-L)**, The relationship between risk-score and age, clinical prognosis stratification, INSS grading, MYCN status and disease aggressiveness were analyzed respectively. *p < 0.1; **p < 0.05; ***p < 0.01, respectively.

To further assess the significance of the 11 genes, we incorporated them into the random forest algorithm. As depicted in **Figure 2D**, we identified the top six genes (TP53, IL-7, PDGFRA, S100B, DLL3, and TP63) to proceed with the subsequent step of the LASSO regression model. The outcomes of the LASSO regression model, including the inclusion of the 6 genes, are presented in **Figures 2E, F**, with the corresponding coefficients assigned to each gene displayed in **Figure 2E**. The expression patterns and levels of these six genes are illustrated in **Figures 3A, E**. Based on the “lambda.min” coefficient shown in **Figure 2F**, all coefficients associated with the six genes are suitable for further analysis. The risk score was calculated using the following formula:

risk score = (0.506 * expression of PDGFRA) + (0.472 * expression of DLL3) + (0.390 * expression of TP53) - (0.598 * expression of S100B) - (0.363 * expression of IL7) - (0.360 * expression of TP63).

### Internal verification of the signature

**Figure 4:**
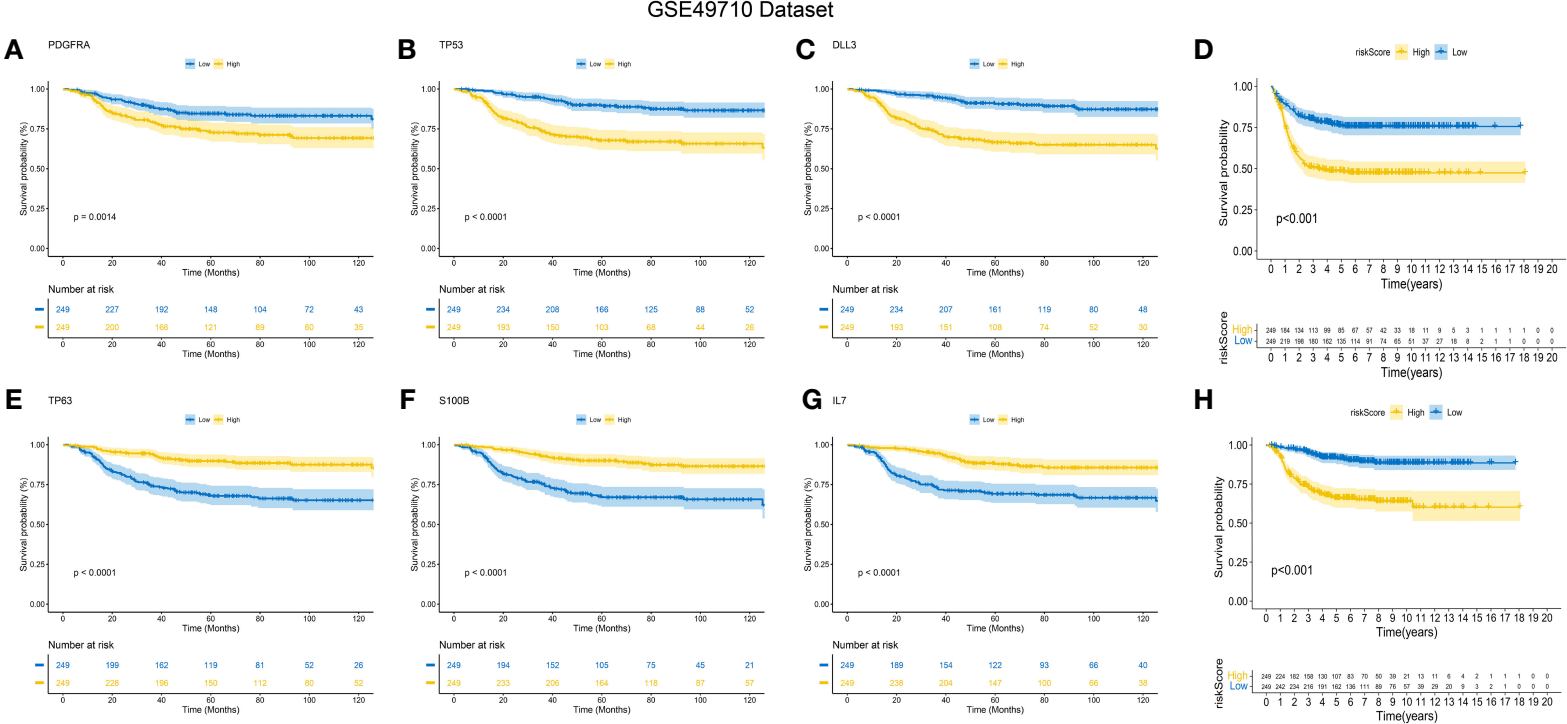
K-M Curve for Prognostic Prediction in NB. **(A-C, E-G)** Survival curves of the relationship between TP53, IL-7, PDGFRA, S100B, DLL3 and TP63 genes and the prognosis of NB patients, respectively. The blue curve indicates low gene expression, while the yellow curve indicates high gene expression. **(D, H)** are the survival curves of EFS and OS of NB patients in the dataset. Blue is the low-risk score, and red is the high-risk score.

The predictive performance of the signature was assessed using Kaplan-Meier survival analysis and the AUC of the ROC curve. The results of the survival analysis demonstrated a close relationship between the six genes included in the model and the prognosis of NB patients, as shown in **Figures 4A-C, E-G**. This finding is consistent with the coefficient trend of the constructed model. Further analysis revealed that patients with low scores had significantly better prognosis in terms of OS or event-free survival (EFS), as depicted in **Figures 4D, H**. The results of the remaining five genes that were not included in the signature are displayed in **Supplementary Figures 1A-E**, respectively. The ROC curve exhibited a high AUC value (AUC=0.968), indicating that the signature possesses excellent predictive ability for OS and EFS (**Figures 3B, C**). During the internal validation process using the GSE49710 dataset, an interesting result emerged: regardless of the 3-year or 5-year EFS or OS, the signature we constructed appeared to have a better fit for predicting the prognosis of NB patients compared to evaluating the prognosis based on MYCN amplification (AUC values of 3 years and 5 years EFS: 0.718 vs. 0.620; 0.709 vs. 0.603, AUC values of 3 years and 5 years OS: 0.824 vs.0.769; 0.793 vs.0.692, respectively), as shown in **Figures 3B, C, F, G**). Another differential analysis revealed that among the 498 NB samples, the group with MYCN amplification had a higher risk score, as illustrated in **Figure 3K** (P<0.0001). This finding is consistent with the differential expression of each gene in the MYCN amplification and non-amplification groups included in the signature, as shown in **Figure 2G** (P<0.0001).

Next, we assessed the relationship between risk scores and age, INSS stages, tumor aggression, and clinical risk stratification. The results indicated that older NB patients had higher genetic ratings for aging (p < 0.001), as depicted in **Figure 3D**. The INSS classification, a commonly used prognostic assessment tool, revealed a higher aging gene score in INSS stage 4 groups compared to others (p < 0.001), as shown in **Figure 3J**. Detailed risk score comparison results are presented in **Figure 3I**. Additionally, clinical high risk and high aggression can be clearly distinguished based on the risk score (p < 0.001), as illustrated in **Figures 3H, L**.

### External validation of the signature

**Figure 5:**
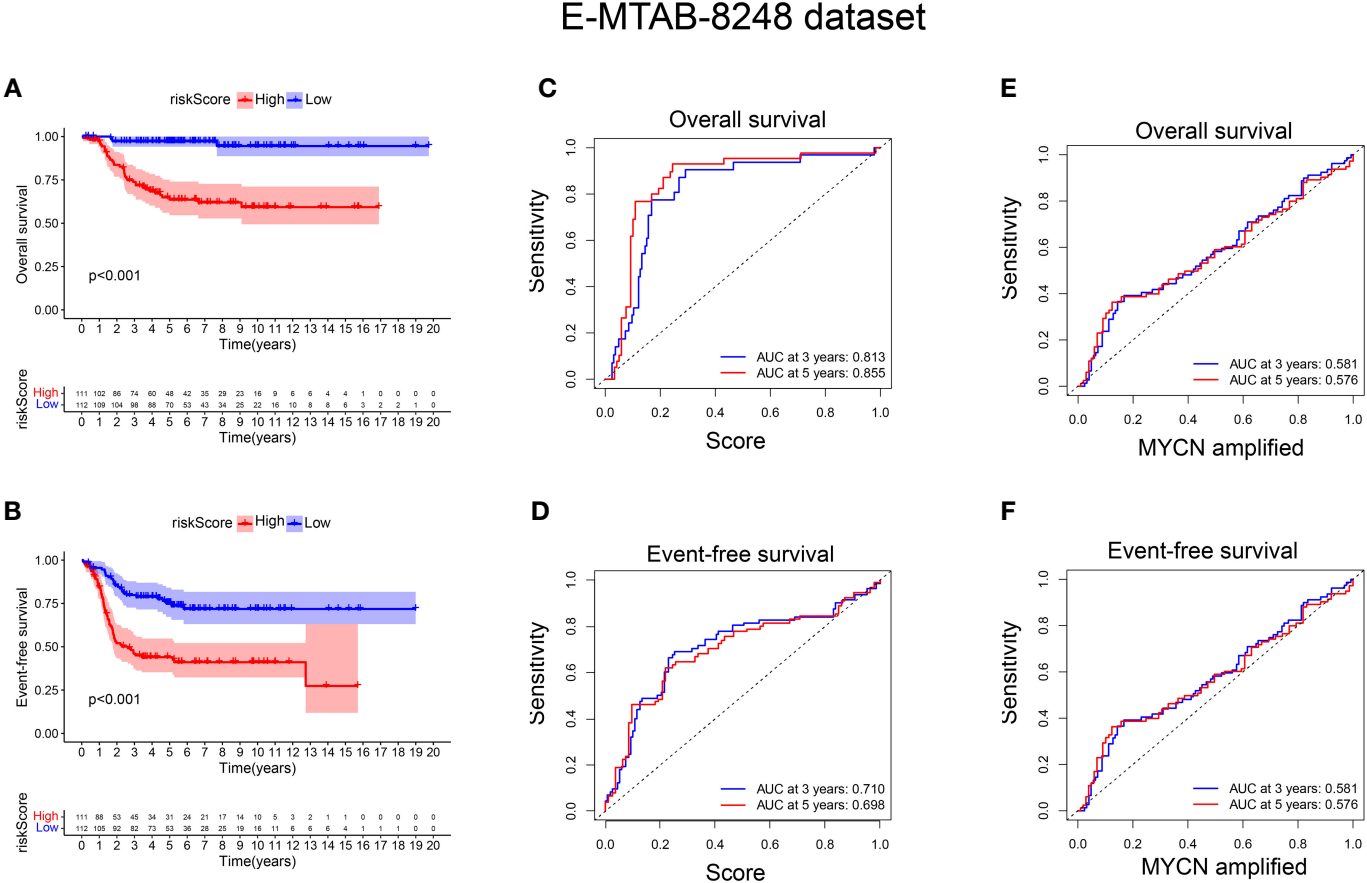
External verification of the relationship between risk score and prognosis of NB patients. **(A, B)** The OS and EFS curves of 223 NB patients were respectively presented, with the blue curve representing patients with low-risk score and the red curve representing patients with high-risk score; **(C, D)** ROC curve shows the value of risk score in evaluating OS and EFS; **(E, F)** ROC curve shows the value of MYCN status in evaluating OS and EFS.

To validate the signature, we utilized the E-MTAB-8248 dataset, which included 223 NB patients. Consistent with the findings from the GSE49710 dataset, the low score group in the E-MTAB-8248 dataset exhibited a significantly better prognosis in terms of OS and EFS compared to the high score group (p < 0.001), as shown in **Figures 5A, B**). Furthermore, our signature demonstrated superior predictive ability for the prognosis of NB patients compared to evaluating prognosis based on MYCN amplification alone, as evidenced by higher AUC values for 3-year and 5-year EFS (0.710 vs. 0.581; 0.698 vs. 0.576) and OS (0.813 vs. 0.581; 0.855 vs. 0.576), as shown in **Figures 5C-F**).

### Positive correlation between risk score, tumor immune microenvironment, and stemness in NB

**Figure 6:**
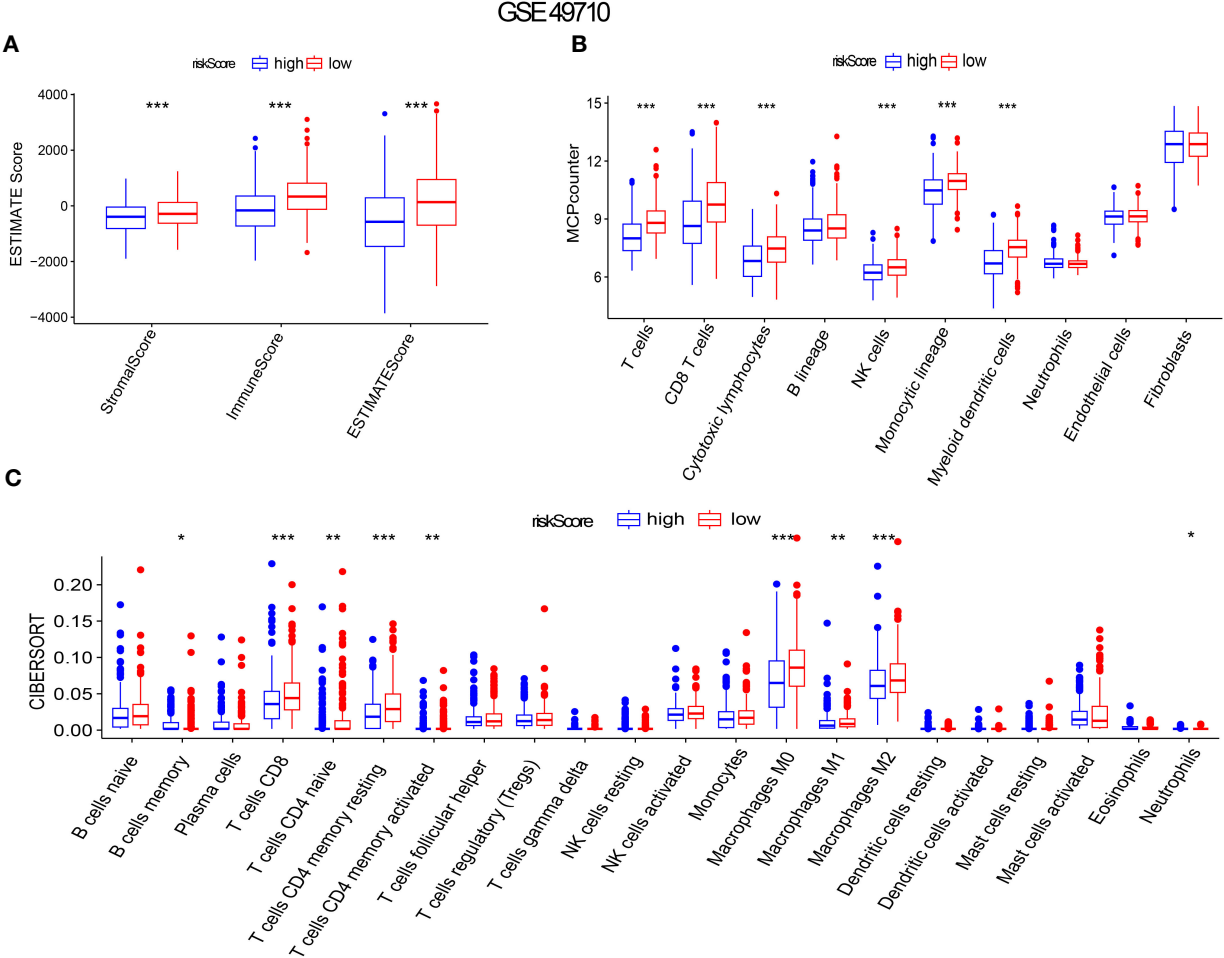
Aging gene score to evaluate immune infiltration in NB tumor microenvironment. **(A-C)** ESTIMATE Score, MICP-counter and CIBERSPORT were used to evaluate immune cell infiltration. The red box represents low-risk score group, while the blue box represents high-risk score group. *p < 0.1; **p < 0.05; ***p < 0.01, respectively.

Gene set enrichment analysis using ESTIMATE effectively captured the presence of stroma in tumor tissue and analyzed immune cell infiltration. Through advanced machine learning techniques, we observed that lower aging gene scores were associated with higher matrix proportion in tumor tissue (p < 0.001), increased immune cell infiltration (p < 0.001), and consequently, lower tumor cell purity (p < 0.001), as shown in **Figure 6A**). We further employed MCP (Microenvironment Cell Populations)-counter to conduct a detailed analysis of immune cell subpopulations in NB tissues. As depicted in **Figure 6B**), NB patients with lower aging gene scores exhibited higher infiltration of T cells, NK cells, myeloid dendritic cells, and monocytes in tumor tissues, with T cells primarily consisting of CD8+ T cells (cytotoxic lymphocytes) (p < 0.001). Additionally, the CIBERSORT algorithm identified four highly infiltrated immune cell subpopulations, namely CD4 naïve T cells, CD4 memory resting T cells, CD4 memory activated T cells, and macrophages, in patients with low scores (p < 0.01, p < 0.001, p < 0.01, p < 0.001, respectively) (**Figure 6C**). **Supplementary Figure 1G** provides a more detailed analysis of immune cell subsets. Tumor cell stemness is closely associated with disease occurrence, drug resistance, recurrence, and metastasis. Correlation analysis revealed a significant positive correlation between aging gene score and tumor cell stemness (R = 0.51, p < 0.001) (**Supplementary Figure 1F**).

### High aging-related prognostic scores are associated with T cell exhaustion and phenotypic changes related to aging

**Figure 7:**
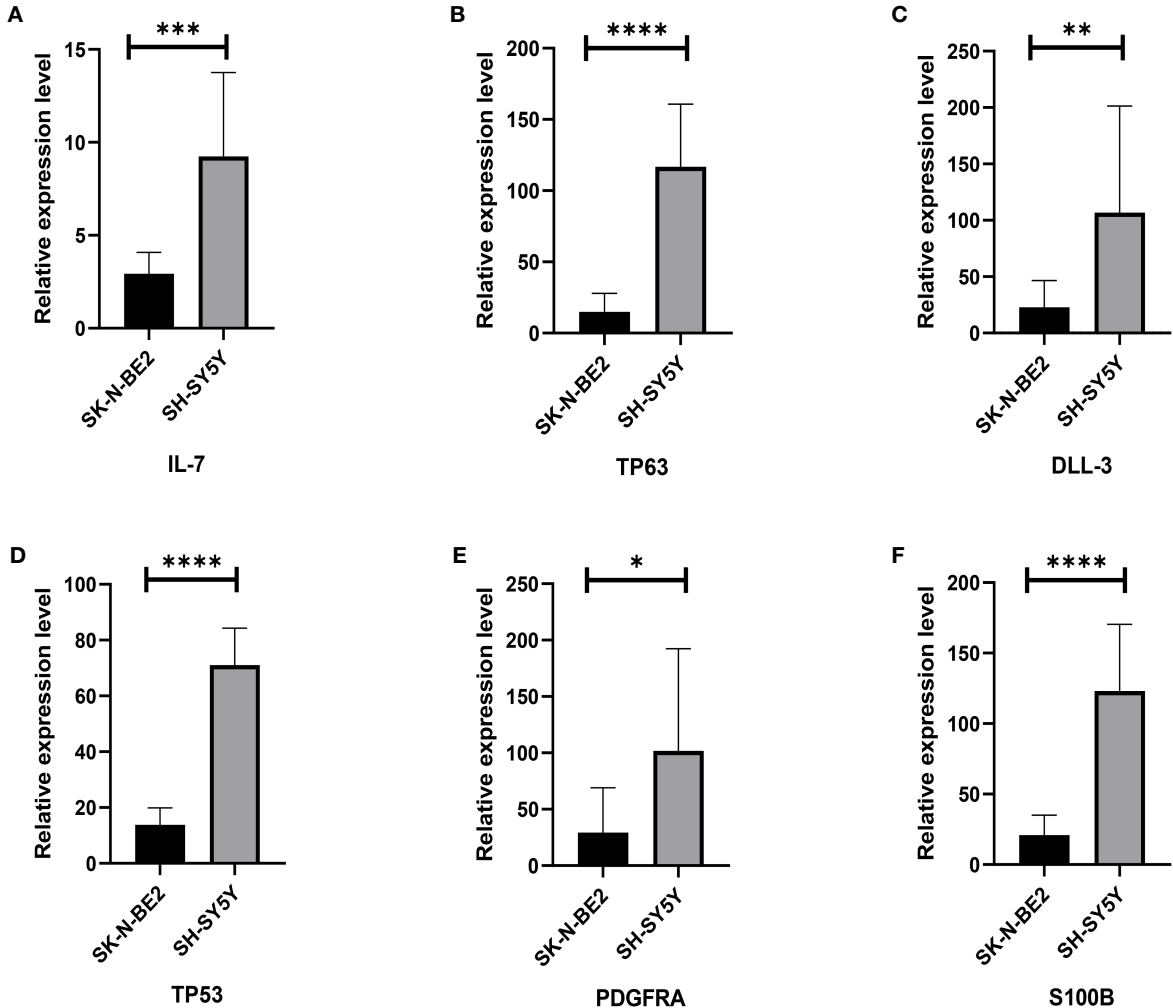
Relative gene expression of 6 included genes A: The **(A–F)** shows qRT-PCR results of IL-7, TP63, DLL3, TP53, PDGFRA and S100B genes in two neuroblastoma cell lines, SH-SY5Y and SK-N-BE (2), respectively. The black column represents the relative expression of gene in SH-SY5Y, and the gray column represents the relative expression of gene in SK-N-BE (2). *p < 0.1; **p < 0.05; ***p < 0.01; ****p < 0.001, respectively.

**Figure 8:**
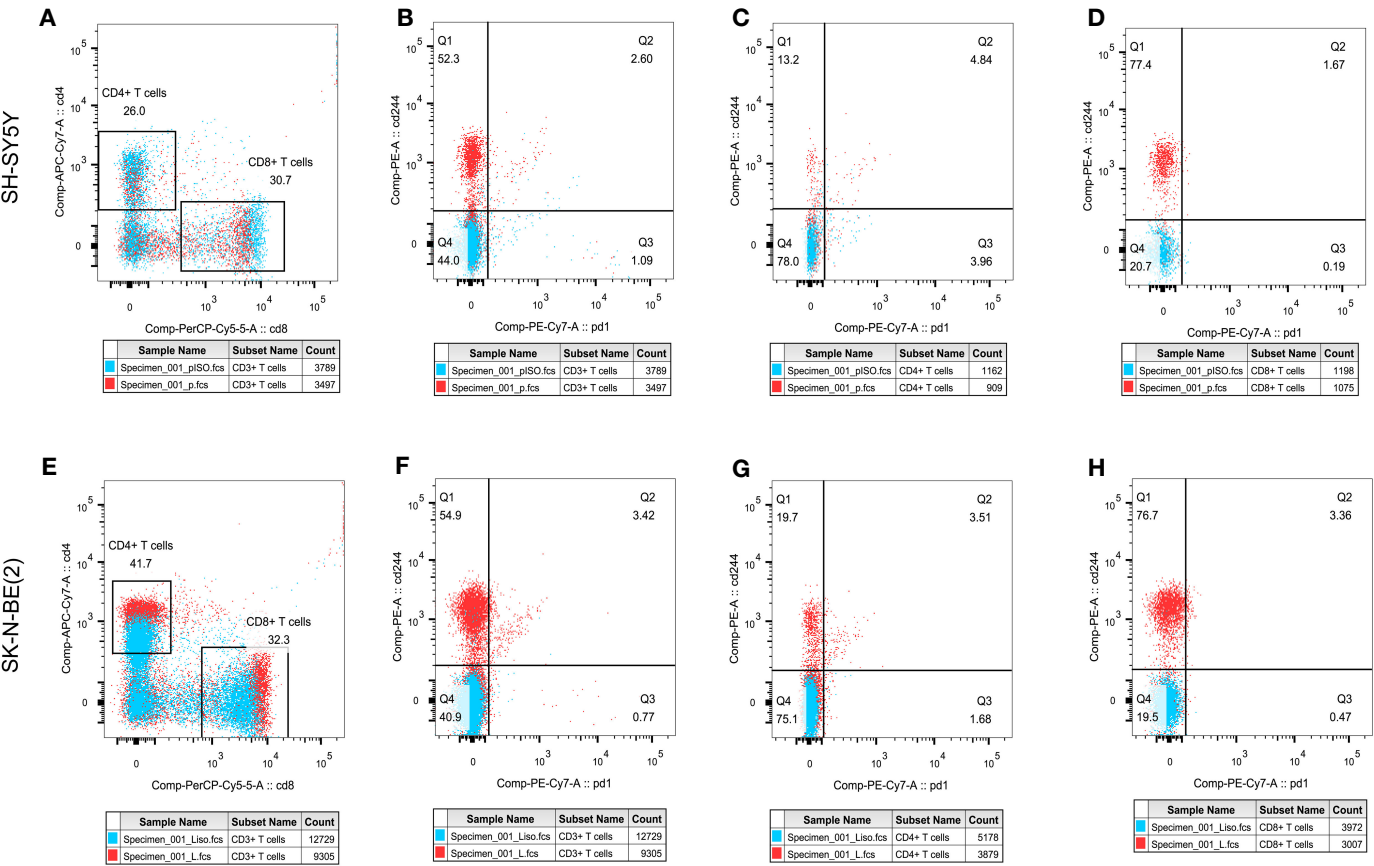
T cell subpopulation distribution and phenotypic changesThe effect of different antigen stimulation on the distribution of T cell subsets and the co-expression of PD-1 and CD244 in different T cell subsets were shown. **(A, E)** show the proportion of CD4+T cell distribution and CD8+T cell distribution after stimulation by SH-SY5Y and SK-N-BE(2), respectively. **(B–D)** reflects the co-expression of CD244 and PD-1 in CD3+, CD4+ and CD8+T cell subsets after SH-SY5Y stimulation. **(F–H)** reflects the co-expression of CD244 and PD-1 in CD3+, CD4+ and CD8+T cell subsets after SK-N-BE(2) stimulation, respectively.

**Figure 9:**
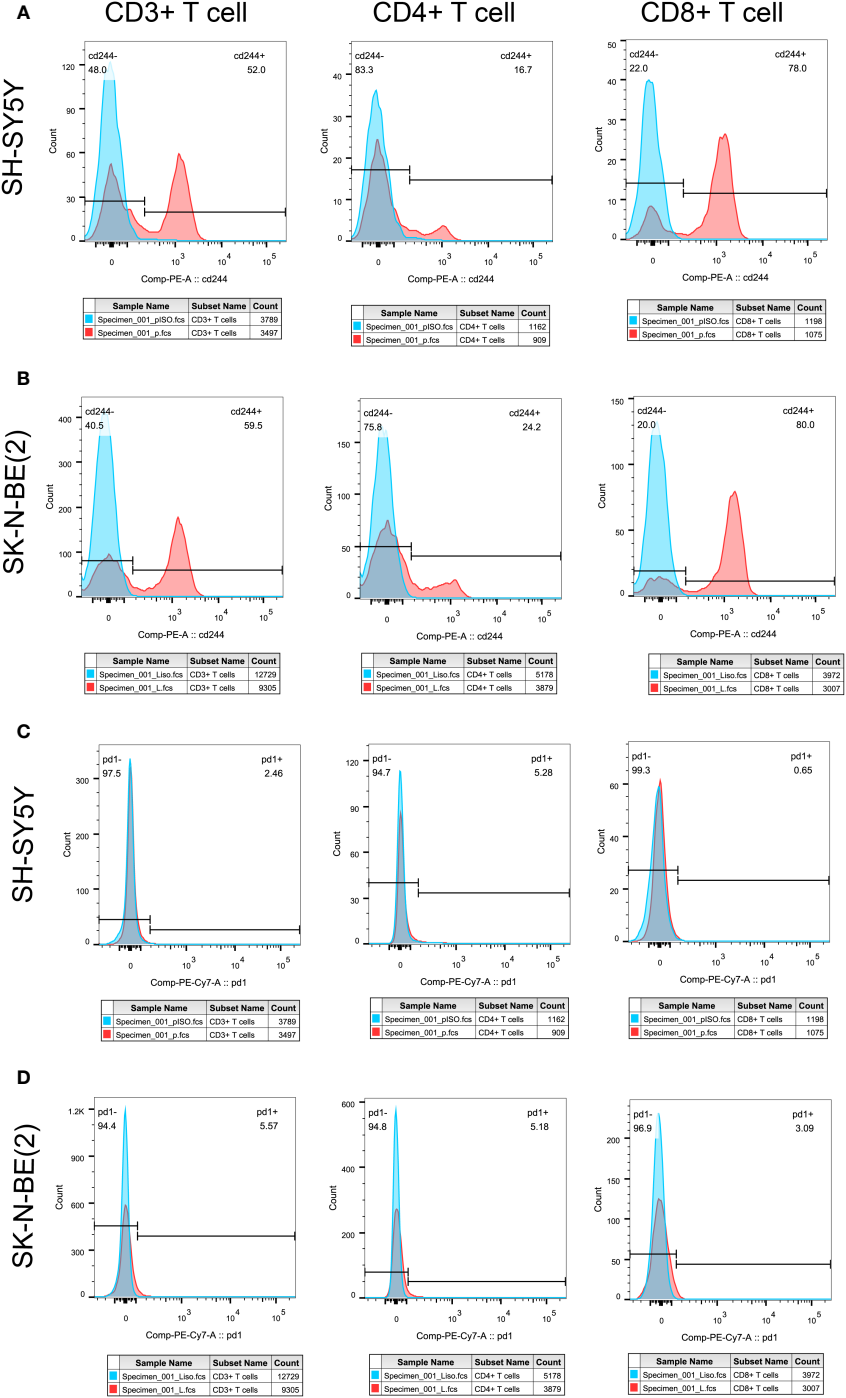
Tumor antigens on T cell exhausted molecules and aging phenotypesT cell exhaustion was shown by the expression ratio of PD-1 molecule, while CD244 was used as the phenotype of T cell senescence. Differential expression of PD-1 and CD244 in CD3+, CD4+ and CD8+T cells were shown. **(A, C)** showed the separate expression of CD244 and PD-1 in CD3+, CD4+, and CD8+T cell subsets after SH-SY5Y stimulation, respectively. **(B, D)** showed the separate expression of CD244 and PD-1 in CD3+, CD4+, and CD8+T cell subsets after SK-N-BE(2) stimulation, respectively. The blue crest comes from the Isotype control, the red crest comes from the fully dyed sample, and all the gates are set according to the Isotype control.

In order to initially validate the impact of our aging-related prognostic scoring system on major T cell subsets, we measured the relative expression levels of six target genes in two NB cell lines with different MYCN amplification, as depicted in **Figures 7A–F**. By applying normalization processing to our constructed model, we calculated the aging prognosis score. The results demonstrated that the SK-N-BE (2) group had a higher aging prognosis score compared to the SH-SY5Y group (risk score: -5.12 vs. -57.45). Next, we employed flow cytometry to assess changes in major T cell subpopulations and phenotypic alterations in PBMCs following contact with NB cells. The findings revealed that the proportion of CD4:CD8 T cells in PBMCs exposed to SK-N-BE (2) was higher than that in the SH-SY5Y group (1.29 vs. 0.85) (**Figure 8**). Further phenotypic analysis indicated that CD8+ T cells exposed to SK-N-BE (2) displayed a higher expression of the exhaustion phenotype marker PD-1, while CD4+ T cells exhibited a higher proportion of the aging phenotype marker CD244 (3.09% vs. 0.65%, 24.2% vs. 16.7%, respectively) (**Figures 9A–D**). Additionally, PBMCs exposed to SK-N-BE (2) showed a higher proportion of the CD244+PD-1+CD8+ T cell subset (3.36% vs. 1.67%) (**Figures 8A–H**).

## Discussion

NB is the most common extracranial tumor in children, and there is currently no standardized prognostic evaluation system, which due to NB is a highly heterogeneous tumor (5, 10). Despite the utilization of genetic testing techniques in clinical practice, only a limited number of genes have established prognostic value in NB (24, 25). It is still unclear why some patients experience poor clinical outcomes despite the absence of commonly detected high-risk genes, while others exhibit positive clinical outcomes despite the presence of individual high-risk gene expression. This discrepancy suggests that there are additional factors beyond the currently recognized high-risk genes that influence the prognosis of NB (25, 26). Redefining the prognostic model of NB based on the expression levels of high-risk genes combined with other mechanisms that may impact prognosis holds significant value. This will ultimately enhance clinical decision-making and optimize patient outcomes.

On the other hand, Prognostic evaluation needs to consider the increasing use of immunotherapy over the past decade, which has been altering the tumor’s prognosis (1, 4). In this study, we constructed six gene composition evaluation models, specifically targeting TP53, PDGFRA, S100B and TP63. These genes are widely recognized as important prognostic indicators in clinical testing and offer the advantages of universality and easy accessibility compared to other pathway models (27–30).

Since the initial description of senescence by Hayflick and Moorhead, our understanding of senescence has continuously evolved (31). The traditional classical theory defines senescence as a state where cells enter permanent cycle arrest while remaining metabolically active in the G0 phase. However, recent studies have challenged this notion and demonstrated that senescence is not necessarily an irreversible state (31). Cells that enter quiescence can still re-enter the replication cycle under certain growth conditions (32, 33). A mouse model study on lymphoma has suggested that senescent cells can be reprogrammed to possess stem cell properties and may re-enter the cell cycle when specific conditions are restored (14). This concept aligns with the definition of tumor cell stemness, which is strongly associated with tumor recurrence and metastasis (14). Our study also found a positive correlation between high senescence scores and tumor cell stemness. Additionally, senescent cells secrete various bioactive cytokines known as SASP (34). SASP may contribute to shaping the immunosuppressive TME, by inducing immune cells to express high senescence phenotype, such as CD244, and exhausted phenotype like PD-1, TIM-3, et al, aiding tumor cell immune escape (11, 35–38). It is important to highlight that senescent tumor cells, upon entering the G0 stage, have the ability to evade the cytotoxic effects of traditional chemotherapy drugs that primarily target actively dividing cells (14). This allows the senescent cells to maintain their survival and potentially contribute to disease progression (14). Considering these findings, it becomes crucial to explore the link between cellular senescence and the inefficacy of immunotherapy in NB.

In a study, cell senescence as an important feature was found in NB samples with MYCN amplification, which is associated with poor prognosis (39). This part of the results aligns with our findings, which indicate a significant difference in the aging score between samples with different MYCN status. Furthermore, we observed a strong correlation between the aging score and prognosis. These findings support the notion that the aging process plays a crucial role in NB progression and can serve as a prognostic indicator (14, 17). The association between MYCN status and aging score suggests that MYCN amplification may contribute to accelerated aging processes in NB cells (40). This could potentially explain the aggressive behavior and poor prognosis associated with MYCN-amplified tumors. Further investigations are warranted to elucidate the underlying mechanisms linking MYCN amplification, aging, and prognosis in NB.

In our subsequent analysis, we focused on examining the correlation between the model constructed based on the six aging-related genes and the prognosis of NB patients. We conducted a comparison of the relative expression levels of six genes in two groups of cell lines with and without MYCN amplification (41). Interestingly, we observed that the relative expression levels of these six high-risk genes were lower in SK-N-BE(2) cells, which are derived from patients with bone marrow metastasis and accompanied by MYCN amplification (41), compared to SH-SY5Y cell lines. This finding is consistent with the clinical observation of lower high-risk gene expression but poor prognosis. On the contrary, using the predictive mode, the SK-N-BE (2) score is indeed much higher than the SH-SY5Y score. This also reflects the fact that our model seems to predict prognosis more accurately and closely to clinical development than analyzing the relative expression of each gene alone. However, further validation with additional clinical data is necessary to confirm these results. It is worth noting that the higher the clinical prognostic stratification of high-risk NB, as indicated by INSS grading, the higher the calculated senescence score in the subgroup with MYCN amplification, shown in **Figure 3**.

Studies on the TME have demonstrated that the formation of an immunosuppressive TME contributes to the poor prognosis of NB patients and the limited effectiveness of immunotherapy (42, 43). In a previous study conducted by our group, we successfully reconstructed normal blood vessels in a mouse model of NB using a multi-target tyrosine kinase inhibitor (TKI) called Anlotinib (9). This approach maximized the efficacy of immunotherapy when combined with a PD-1 inhibitor, highlighting the crucial role of the TME in immunotherapy (9). NB has been recognized as an excellent model for studying cold tumors, making the analysis of immune cell infiltration within NB tumor tissues vital for understanding the TME (44). By analyzing the composition of tumor tissues in different senescence score subgroups, we observed that high-scoring NB tumor tissues exhibited lower immune scores and stromal scores, indicating a lower percentage of immune cells. Further subpopulation analysis revealed significantly reduced infiltration of CD8+ T cells, NK cells, myeloid dendritic cells, monocytes, and macrophages in high-scoring NB tissues. These findings are consistent with studies that decreased immune cell infiltration in the TME of NB patients with poor prognosis and ineffective immunotherapy (45, 46). CD244 (2B4) binding to the ligand CD48 has been found to be a signaling pathway for co-stimulation or negative regulation of multiple immune cells in tumor, that currently considered to be an important marker of immune cell senescence (47, 48). CD244+ CD8+ aging T cells exhibited features of exhaustion, including lower levels of cytokine, impaired proliferation, and intrinsic transcriptional regulation (49). Phenotype analysis showed that the proportion of CD8+ T cells did not increase after PBMC exposure to SK-N-BE (2) tumor antigen as it did after exposure to SH-SY5Y tumor antigen. What’s even more interesting is that CD8+ T cells exposed to SK-N-BE (2) displayed a higher expression of the exhaustion phenotype marker PD-1, while CD4+ T cells exhibited a higher proportion of the aging phenotype marker CD244 were found. Additionally, PBMCs exposed to SK-N-BE (2) showed a higher proportion of the CD244+PD-1+CD8+ T cell subset. The increase in the proportion of CD244+PD-1+ T cells was found in acute myeloid leukemia and non-Hodgkin’s lymphoma significantly, that may be related to the occurrence and development of tumor (50–52). This indicate that CD8+ T cells exposed to SK-N-BE (2) antigen may enter a state of functional exhaustion and cellular senescence.

However, it is important to acknowledge the limitations of our study. Firstly, considering the addition of cytokines and cell-activating antibodies in our experiments, we did not specifically measure cytokine levels. To address this limitation, we plan to use plasma samples from NB patients in future experiments to investigate the SASP. Additionally, the operability and prognostic value of this model should be verified through extensive clinical practice. By addressing these limitations and conducting additional research, we aim to strengthen the validity and applicability of our findings. Ultimately, our goal is to contribute to the advancement of NB prognosis prediction and guide personalized treatment strategies for improved patient outcomes.

## Conclusion

In summary, this study focused on identifying MYCN-related differential genes and senescence molecules in NB patients. The researchers constructed a six-gene signature and validated its predictive ability for the prognosis of NB patients. The signature was also found to be associated with the tumor immune microenvironment and stemness in NB.

## Data availability statement

The datasets presented in this study can be found in online repositories. The names of the repository/repositories and accession number(s) can be found in the article/**Supplementary Material**.

## Ethics statement

Ethical approval was not required for the studies on animals in accordance with the local legislation and institutional requirements because only commercially available established cell lines were used.

## Author contributions

JT: Data curation, Formal Analysis, Methodology, Validation, Writing – original draft, Writing – review & editing. CW: Data curation, Software, Writing – review & editing. YJ: Conceptualization, Formal Analysis, Writing – review & editing. YX: Funding acquisition, Writing – review & editing. BG: Investigation, Software, Writing – review & editing. QZ: Formal Analysis, Funding acquisition, Supervision, Writing – review & editing.

## Supplementary material

Supplementary Figure 1 The relationship between aging genes and prognosis, tumor cell stemness and immune invasion. **(A-E)** The remaining five genes that were not included in the signature was associated with poor OS in GSE49710 datasets. **(F)** Correlation analysis between risk score and the stemness of NB tumor cell. **(G)** xCell method was used to analyze the relationship between risk score and immune cell infiltration in NB tumor tissue. *p < 0.1; **p < 0.05; ***p < 0.01; ****p < 0.001, respectively.

## References

[1] Anderson J Majzner RG Sondel PM. Immunotherapy of neuroblastoma: facts and hopes. Clin Cancer Res (2022) 28(15):3196–206. doi: 10.1158/1078-0432.CCR-21-1356 PMC9344822 35435953

[2] Liu Z Yu X Xu L Li Y Zeng C. Current insight into the regulation of PD-L1 in cancer. Exp Hematol Oncol (2022) 11(1):44. doi: 10.1186/s40164-022-00297-8 35907881 PMC9338491

[3] Alborzinia H Florez AF Kreth S Bruckner LM Yildiz U Gartlgruber M. MYCN mediates cysteine addiction and sensitizes neuroblastoma to ferroptosis. Nat Cancer (2022) 3(4):471–85. doi: 10.1038/s43018-022-00355-4 PMC9050595 35484422

[4] Zafar A Wang W Liu G Wang X Xian W McKeon F. Molecular targeting therapies for neuroblastoma: Progress and challenges. Medicinal Res Rev (2021) 41(2):961–1021. doi: 10.1002/med.21750 PMC7906923 33155698

[5] Croteau N Nuchtern J LaQuaglia MP. Management of neuroblastoma in pediatric patients. Surg Oncol Clinics North America (2021) 30(2):291–304. doi: 10.1016/j.soc.2020.11.010 33706901

[6] Wagner LM Adams VR. Targeting the PD-1 pathway in pediatric solid tumors and brain tumors. OncoTargets Ther (2017) 10:2097–106. doi: 10.2147/OTT.S124008 PMC5396947 28442918

[7] Camus M Tosolini M Mlecnik B Pages F Kirilovsky A Berger A. Coordination of intratumoral immune reaction and human colorectal cancer recurrence. Cancer Res (2009) 69(6):2685–93. doi: 10.1158/0008-5472.CAN-08-2654 19258510

[8] El-Hajjar M Gerhardt L Hong MMY Krishnamoorthy M Figueredo R Zheng X. Inducing mismatch repair deficiency sensitizes immune-cold neuroblastoma to anti-CTLA4 and generates broad anti-tumor immune memory. Mol Ther J Am Soc Gene Ther (2023) 31(2):535–51. doi: 10.1016/j.ymthe.2022.08.025 PMC9931548 36068918

[9] Su Y Luo B Lu Y Wang D Yan J Zheng J. Anlotinib induces a T cell-inflamed tumor microenvironment by facilitating vessel normalization and enhances the efficacy of PD-1 checkpoint blockade in neuroblastoma. Clin Cancer Res (2022) 28(4):793–809. doi: 10.1158/1078-0432.CCR-21-2241 34844980 PMC9377760

[10] Wienke J Dierselhuis MP Tytgat GAM Kunkele A Nierkens S Molenaar JJ. The immune landscape of neuroblastoma: Challenges and opportunities for novel therapeutic strategies in pediatric oncology. Eur J Cancer (2021) 144:123–50. doi: 10.1016/j.ejca.2020.11.014 33341446

[11] Zhong M Gao R Zhao R Huang Y Chen C Li K. BET bromodomain inhibition rescues PD-1-mediated T-cell exhaustion in acute myeloid leukemia. Cell Death Dis (2022) 13(8):671. doi: 10.1038/s41419-022-05123-x 35918330 PMC9346138

[12] Alleboina S Aljouda N Miller M Freeman KW. Therapeutically targeting oncogenic CRCs facilitates induced differentiation of NB by RA and the BET bromodomain inhibitor. Mol Ther oncolytics (2021) 23:181–91. doi: 10.1016/j.omto.2021.09.004 PMC8526497 34729395

[13] Hayflick L Moorhead PS. The serial cultivation of human diploid cell strains. Exp Cell Res (1961) 25:585–621. doi: 10.1016/0014-4827(61)90192-6 13905658

[14] Milanovic M Fan DNY Belenki D Dabritz JHM Zhao Z Yu Y. Senescence-associated reprogramming promotes cancer stemness. Nature (2018) 553(7686):96–100. doi: 10.1038/nature25167 29258294

[15] Saleh T Tyutyunyk-Massey L Murray GF Alotaibi MR Kawale AS Elsayed Z. Tumor cell escape from therapy-induced senescence. Biochem Pharmacol (2019) 162:202–12. doi: 10.1016/j.bcp.2018.12.013 30576620

[16] Wiley CD Velarde MC Lecot P Liu S Sarnoski EA Freund A. Mitochondrial dysfunction induces senescence with a distinct secretory phenotype. Cell Metab (2016) 23(2):303–14. doi: 10.1016/j.cmet.2015.11.011 PMC4749409 26686024

[17] Sager R. Senescence as a mode of tumor suppression. Environ Health Perspect (1991) 93:59–62. doi: 10.1289/ehp.919359 1663451 PMC1568048

[18] Lin W Wang X Wang Z Shao F Yang Y Cao Z. Comprehensive analysis uncovers prognostic and immunogenic characteristics of cellular senescence for lung adenocarcinoma. Front Cell Dev Biol (2021) 9:780461. doi: 10.3389/fcell.2021.780461 34869385 PMC8636167

[19] Tubita A Lombardi Z Tusa I Lazzeretti A Sgrignani G Papini D. Inhibition of ERK5 Elicits Cellular Senescence in Melanoma via the Cyclin-Dependent Kinase Inhibitor p21. Cancer Res (2022) 82(3):447–57. doi: 10.1158/0008-5472.CAN-21-0993 PMC9397638 34799355

[20] Yoshihara K Shahmoradgoli M Martinez E Vegesna R Kim H Torres-Garcia W. Inferring tumour purity and stromal and immune cell admixture from expression data. Nat Commun (2013) 4:2612. doi: 10.1038/ncomms3612 24113773 PMC3826632

[21] Newman AM Liu CL Green MR Gentles AJ Feng W Xu Y. Robust enumeration of cell subsets from tissue expression profiles. Nat Methods (2015) 12(5):453–7. doi: 10.1038/nmeth.3337 PMC4739640 25822800

[22] Becht E Giraldo NA Lacroix L Buttard B Elarouci N Petitprez F. Estimating the population abundance of tissue-infiltrating immune and stromal cell populations using gene expression. Genome Biol (2016) 17(1):218. doi: 10.1186/s13059-016-1070-5 27765066 PMC5073889

[23] Aran D Hu Z Butte AJ. xCell: digitally portraying the tissue cellular heterogeneity landscape. Genome Biol (2017) 18(1):220. doi: 10.1186/s13059-017-1349-1 29141660 PMC5688663

[24] Tian XM Xiang B Yu YH Li Q Zhang ZX Zhanghuang C. A novel cuproptosis-related subtypes and gene signature associates with immunophenotype and predicts prognosis accurately in neuroblastoma. Front Immunol (2022) 13:999849. doi: 10.3389/fimmu.2022.999849 36211401 PMC9540510

[25] Fetahu IS Taschner-Mandl S. Neuroblastoma and the epigenome. Cancer metastasis Rev (2021) 40(1):173–89. doi: 10.1007/s10555-020-09946-y PMC7897201 33404859

[26] Chen G Luo D Zhong N Li D Zheng J Liao H. GPC2 is a potential diagnostic, immunological, and prognostic biomarker in pan-cancer. Front Immunol (2022) 13:857308. doi: 10.3389/fimmu.2022.857308 35345673 PMC8957202

[27] Wang H Wang X Xu L Zhang J. TP53 and TP53-associated genes are correlated with the prognosis of paediatric neuroblastoma. BMC genomic Data (2022) 23(1):41. doi: 10.1186/s12863-022-01059-5 35655142 PMC9164562

[28] Mei Y Wang Z Zhang L Zhang Y Li X Liu H. Regulation of neuroblastoma differentiation by forkhead transcription factors FOXO1/3/4 through the receptor tyrosine kinase PDGFRA. Proc Natl Acad Sci USA (2012) 109(13):4898–903. doi: 10.1073/pnas.1119535109 PMC3323967 22411791

[29] Bernardini C Lattanzi W Businaro R Leone S Corvino V Sorci G. Transcritpional effects of S100B on neuroblastoma cells: perturbation of cholesterol homeostasis and interference on the cell cycle. Gene Expression (2010) 14(6):345–59. doi: 10.3727/105221610X12718619643013 PMC6042022 20635576

[30] Levrero M De Laurenzi V Costanzo A Gong J Wang JY Melino G. The p53/p63/p73 family of transcription factors: overlapping and distinct functions. J Cell Sci (2000) 113(Pt 10):1661–70. doi: 10.1242/jcs.113.10.1661 10769197

[31] de Magalhaes JP Passos JF. Stress, cell senescence and organismal ageing. Mech Ageing Dev (2018) 170:2–9. doi: 10.1016/j.mad.2017.07.001 28688962

[32] Foster DA Yellen P Xu L Saqcena M. Regulation of G1 cell cycle progression: distinguishing the restriction point from a nutrient-sensing cell growth checkpoint(s). Genes Cancer (2010) 1(11):1124–31. doi: 10.1177/1947601910392989 PMC3092273 21779436

[33] Liu J Wang L Wang Z Liu JP. Roles of telomere biology in cell senescence, replicative and chronological ageing. Cells (2019) 8(1):54. doi: 10.3390/cells8010054 30650660 PMC6356700

[34] Birch J Gil J. Senescence and the SASP: many therapeutic avenues. Genes Dev (2020) 34(23-24):1565–76. doi: 10.1101/gad.343129.120 PMC7706700 33262144

[35] Giroud J Bouriez I Paulus H Pourtier A Debacq-Chainiaux F Pluquet O. Exploring the communication of the SASP: dynamic, interactive, and adaptive effects on the microenvironment. Int J Mol Sci (2023) 24(13):10788. doi: 10.3390/ijms241310788 37445973 PMC10341516

[36] Mabrouk N Ghione S Laurens V Plenchette S Bettaieb A Paul C. Senescence and cancer: role of nitric oxide (NO) in SASP. Cancers (2020) 12(5):1145. doi: 10.3390/cancers12051145 32370259 PMC7281185

[37] Takasugi M Yoshida Y Ohtani N. Cellular senescence and the tumour microenvironment. Mol Oncol (2022) 16(18):3333–51. doi: 10.1002/1878-0261.13268 PMC9490140 35674109

[38] Agresta L Hoebe KHN Janssen EM. The emerging role of CD244 signaling in immune cells of the tumor microenvironment. Front Immunol (2018) 9:2809. doi: 10.3389/fimmu.2018.02809 30546369 PMC6279924

[39] Ackermann S Cartolano M Hero B Welte A Kahlert Y Roderwieser A. A mechanistic classification of clinical phenotypes in neuroblastoma. Science (2018) 362(6419):1165–70. doi: 10.1126/science.aat6768 PMC7875194 30523111

[40] Matthay KK Maris JM Schleiermacher G Nakagawara A Mackall CL Diller L. Neuroblastoma. Nat Rev Dis Primers (2016) 2:16078. doi: 10.1038/nrdp.2016.78 27830764

[41] Hossain MM Banik NL Ray SK. Survivin knockdown increased anti-cancer effects of (-)-epigallocatechin-3-gallate in human Malignant neuroblastoma SK-N-BE2 and SH-SY5Y cells. Exp Cell Res (2012) 318(13):1597–610. doi: 10.1016/j.yexcr.2012.03.033 PMC3374045 22507272

[42] Sha YL Liu Y Yang JX Wang YY Gong BC Jin Y. B3GALT4 remodels the tumor microenvironment through GD2-mediated lipid raft formation and the c-met/AKT/mTOR/IRF-1 axis in neuroblastoma. J Exp Clin Cancer Res CR (2022) 41(1):314. doi: 10.1186/s13046-022-02523-x 36284313 PMC9594894

[43] Rivera Z Escutia C Madonna MB Gupta KH. Biological insight and recent advancement in the treatment of neuroblastoma. Int J Mol Sci (2023) 24(10):8470. doi: 10.3390/ijms24108470 37239815 PMC10218416

[44] Voeller J Erbe AK Slowinski J Rasmussen K Carlson PM Hoefges A. Combined innate and adaptive immunotherapy overcomes resistance of immunologically cold syngeneic murine neuroblastoma to checkpoint inhibition. J immunotherapy Cancer (2019) 7(1):344. doi: 10.1186/s40425-019-0823-6 PMC6898936 31810498

[45] Sherif S Roelands J Mifsud W Ahmed EI Raynaud CM Rinchai D. The immune landscape of solid pediatric tumors. J Exp Clin Cancer Res CR (2022) 41(1):199. doi: 10.1186/s13046-022-02397-z 35690832 PMC9188257

[46] Vanichapol T Chutipongtanate S Anurathapan U Hongeng S. Immune escape mechanisms and future prospects for immunotherapy in neuroblastoma. BioMed Res Int (2018) 2018:1812535. doi: 10.1155/2018/1812535 29682521 PMC5845499

[47] Sun L Gang X Li Z Zhao X Zhou T Zhang S. Advances in understanding the roles of CD244 (SLAMF4) in immune regulation and associated diseases. Front Immunol (2021) 12:648182. doi: 10.3389/fimmu.2021.648182 33841431 PMC8024546

[48] Pita-Lopez ML Gayoso I DelaRosa O Casado JG Alonso C Munoz-Gomariz E. Effect of ageing on CMV-specific CD8 T cells from CMV seropositive healthy donors. Immun Ageing I A (2009) 6:11. doi: 10.1186/1742-4933-6-11 19715573 PMC2741428

[49] Wang X Wang D Du J Wei Y Song R Wang B. High levels of CD244 rather than CD160 associate with CD8(+) T-cell aging. Front Immunol (2022) 13:853522. doi: 10.3389/fimmu.2022.853522 35386693 PMC8977780

[50] Tan J Yu Z Huang J Chen Y Huang S Yao D. Increased PD-1+Tim-3+ exhausted T cells in bone marrow may influence the clinical outcome of patients with AML. biomark Res (2020) 8:6. doi: 10.1186/s40364-020-0185-8 32082573 PMC7020501

[51] Huang S Liang C Zhao Y Deng T Tan J Zha X. Increased TOX expression concurrent with PD-1, Tim-3, and CD244 expression in T cells from patients with acute myeloid leukemia. Cytometry Part B Clin cytometry (2022) 102(2):143–52. doi: 10.1002/cyto.b.22049 34913594

[52] Huang S Liang C Zhao Y Deng T Tan J Lu Y. Increased TOX expression concurrent with PD-1, Tim-3, and CD244 in T cells from patients with non-Hodgkin lymphoma. Asia-Pacific J Clin Oncol (2022) 18(1):143–9. doi: 10.1111/ajco.13545 33608984

