# Supplementary material for: Optimal combination of MYCN differential gene and cellular senescence gene predicts adverse outcomes in patients with neuroblastoma
Source: Front Immunol. 2023 Nov 16;14:1309138. doi: 10.3389/fimmu.2023.1309138 (PMC10687280; doi:10.3389/fimmu.2023.1309138)
Supplement: Supplementary file 1 [file Table_1.docx]

**Supplementary Table 1. Six target gene primers and internal reference primers**

| **Gene** | **Forward primer** | **Reverse primer** |
| --- | --- | --- |
| TP53 | AGTCTAGAGCCACCGTCCAG | ACAGTCAGAGCCAACCTCAG |
| TP63 | CCCTGACCCTTACATCCAGC | CAGGACTTGCCCATCTCTGG |
| IL-7 | GCCAAGGCGTTGAGAGATCA | TGGTTTTCTTCCTTTAACCTGGC |
| PDGFRA | GGGCACGCTCTTTACTCCAT | TTAGGCTCAGCCCTGTGAGA |
| DLL3 | AGGCAGCTGTAGTGAGACAC | AGGGTAGGGAAAAAGCAGGTG |
| S100B | GGTGAGACAAGGAAGAGGATGT | CCTCCGGGTTAGGGTCTACA |
| GAPDH | CAAGGTCATCCATGACAACTTTG | GTCCACCACCCTGTTGCTGTAG |
