# Supplementary figures and images for: Optimal combination of MYCN differential gene and cellular senescence gene predicts adverse outcomes in patients with neuroblastoma
Source: Front Immunol. 2023 Nov 16;14:1309138. doi: 10.3389/fimmu.2023.1309138 (PMC10687280; doi:10.3389/fimmu.2023.1309138)

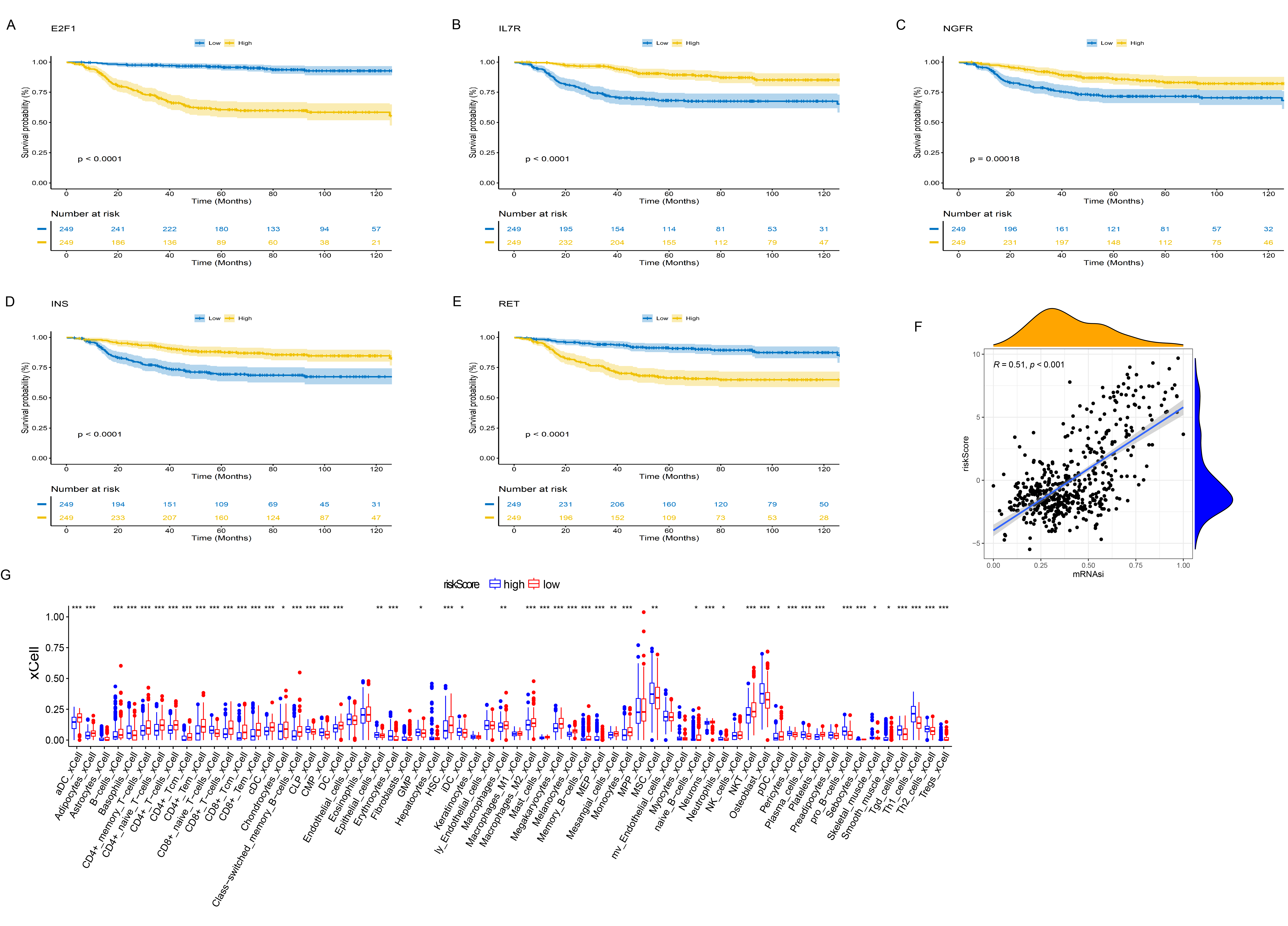

Supplement: Supplementary Figure 1 — The relationship between aging genes and prognosis, tumor cell stemness and immune invasion. (A-E) The remaining five genes that were not included in the signature was associated with poor OS in GSE49710 datasets. (F) Correlation analysis between risk score and the stemness of NB tumor cell. (G) xCell method was used to analyze the relationship between risk score and immune cell infiltration in NB tumor tissue. *p < 0.1; **p < 0.05; ***p < 0.01; ****p < 0.001, respectively. [file Image_1.tif]
